# Migrated toothpick causing a hepatic abscess with portal vein thrombosis: A case report and review of literature

**DOI:** 10.1002/ccr3.9332

**Published:** 2024-08-21

**Authors:** Faisal Joueidi, Ali A. Alzahrani, Abdulaziz A. Altaweel, Omar Alwhaibi, Ahmed Elgohary, Khalid O. Bin Saad

**Affiliations:** ^1^ College of Medicine Alfaisal University Riyadh Riyadh Saudi Arabia; ^2^ College of Medicine King Saud University Riyadh Saudi Arabia; ^3^ King Faisal Specialist Hospital and Research Centre Riyadh Saudi Arabia; ^4^ College of Medicine Imam Muhammad Bin Saud Islamic University Riyadh Saudi Arabia; ^5^ Department of Hepatobiliary Sciences King Abdulaziz Medical City Riyadh Saudi Arabia

**Keywords:** case report, foreign body, hepatic, liver abscess, portal vein thrombosis, toothpick

## Abstract

**Key Clinical Message:**

Accidental foreign body ingestion is the most common hidden cause of abdominal pain. A high index of suspicion should be implemented in patients with unresolved abdominal pain. Here we reported a 54‐year‐old patient with vague abdominal pain who had a successful laparoscopic removal of a toothpick.

**Abstract:**

Toothpicks and fish bones are considered one of the most common accidentally ingested foreign bodies. Fortunately, most patients are asymptomatic. About 80%–90% of ingested foreign bodies pass through the gut spontaneously within a week. We present a case of a 54‐year‐old female with chronic epigastric pain and fever found to have a foreign body (toothpick) that penetrated the stomach and migrated to the liver causing liver abscess with portal vein thrombosis. The patient was managed with laparoscopic removal of the foreign body with an uneventful postoperative course.

## 1 INTRODUCTION

Sharp foreign bodies (toothpicks, fish bones, needles, chicken bone, etc.) are infrequently ingested accidentally. Also, patients may present with critical conditions such as abscess formation, visceral perforation, visceral inflammation, or simply mild irritation to the gastrointestinal tract related to the site of penetration.^1^, ^2^ Fortunately, most patients are asymptomatic and the foreign body will pass spontaneously within a week.^3^ The various clinical presentations of the patients, the absence of awareness of ingesting the foreign body, and the site of penetration increase the difficulty of clinical diagnosis.^4^ It has been found that <1% of ingested foreign bodies cause gastrointestinal perforation, especially in the ileocecal junction and rectosigmoid region.^5^, ^6^, ^7^ These patients usually come with abdominal pain and fever, and some will present with a full picture of the acute abdomen.^8^ Unfortunately, other differential diagnosis such as acute cholecystitis, pancreatic carcinoma, and peptic ulcer disease may mislead the medical team.^9^, ^10^, ^11^ Moreover, Goh et al.^12^ reported that only 23% of total ingested foreign bodies cases were diagnosed pre‐operatively.^12^ Our reported case focuses on a foreign body (toothpick) penetrating the gastric wall and migrating to the liver causing left hepatic abscess and portal vein thrombosis. Previous literature regarding foreign body ingestion is rare, the first case was reported by Lambert.^13^ In 2012, Abu‐Wasel et al.^14^ reported the presence of 17 worldwide cases of hepatic abscess formation secondary to toothpick formation.^14^ A literature review of portal vein thrombosis sequelae to foreign body migration causing hepatic abscess has not yet been reported.

## 2 CASE REPORT

### 2.1 History and physical examination

A 54‐year‐old female presented on June 11, 2017, to King Abdulaziz Medical City Riyadh's emergency department with chronic dull epigastric pain worsening lately and started to be associated with fever. The patient denied any history of nausea, vomiting, anorexia, or change in bowel habits. This condition started 2 months back with vague abdominal pain managed initially with painkillers and antibiotics with several admissions to local hospitals. Physical examination was unremarkable.

### 2.2 Laboratory Investigations

Basic Labs were done: WBC of 5.6×10^9/L, low hemoglobin of 88 gm/L, high platelet count of 466×10^9/L, high alkaline phosphatase of 294 U/L, normal levels of aspartate aminotransferase of 30 and alanine transaminase (ALT) of 30, high GGT of 133 U/L, total bilirubin of 6 μmol/L, low creatinine levels of 47 μmol/L, and normal coagulation profile.

### 2.3 Radiological investigations

**FIGURE 1:**
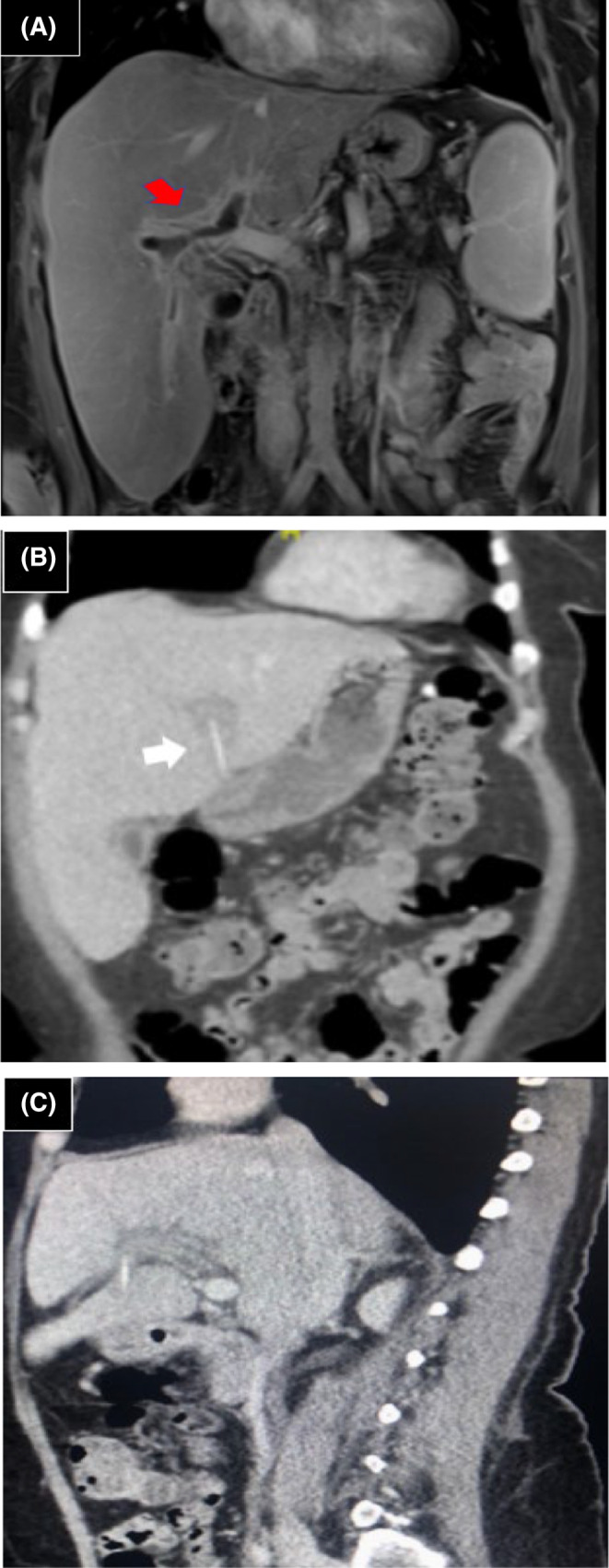
(A) Coronal (red arrow) MRI shows left, right, and upper main portal vein thrombophlebitis associated with inflammatory changes, (B) Coronal (white arrow) and (C) sagittal CT views demonstrated a 3.5 cm liner hyperdense structure.

CT scans of the abdomen showed a linear hyperdense structure in the left lobe of the liver with thrombosis of the main portal vein along with the main right and the main left branches. MRI scans of the abdomen showed a foreign body in segment three of the left lobe of the liver associated with intrahepatic abscess formation and clear tinting of stomach serosa representing the site of foreign body penetration. At that time, she was managed conservatively with antibiotics and heparin. On June 12, 2017, repeated CT scans of the abdominal and pelvis demonstrated a 3.5 cm foreign body of linear hyperdensity configuration penetrating the gastric pylorus with the tip remaining inside the stomach (Figure 1). The foreign body had migrated to the intrahepatic fissure. The left portal vein was found to be thrombosed with a resolution of the main and the right portal vein thrombosis. There was no active abscess (resolved).On June 14, 2017, the patient underwent an upper GI endoscopy, and no foreign body was found in the stomach, pylorus, or first part of the duodenum.

### 2.4 Final diagnosis

Migrated toothpick complicated by a Hepatic Abscess and Portal Vein Thrombosis.

### 2.5 Management

**FIGURE 2:**
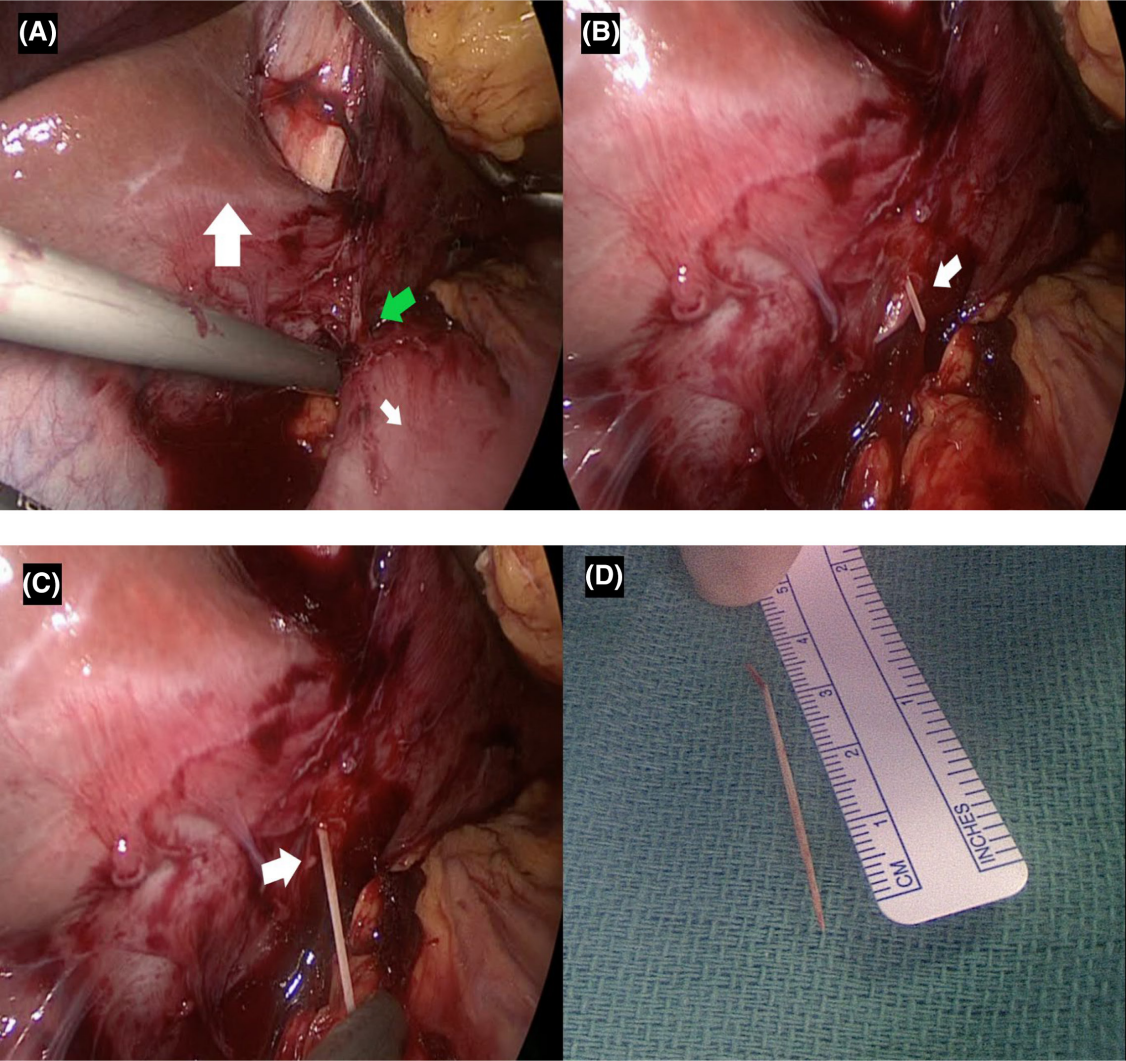
(A) Fibrosis and adhesions seen around the foreign body (green arrow) penetrating left lobe of liver (large white arrow) from the stomach (small white arrow). (B) Part of foreign body (white arrow) becomes apparent after breaking the adhesions between liver and stomach. (C) Pulling the toothpick (white arrow) out of the left liver lobe. (D) Toothpick after final removal with approximate length of 4 cm.

The patient was started on Meropenem and Enoxaparin. The plan was to proceed with surgery. On June 19, 2017, the patient was taken to the operating room and underwent a 4‐port laparoscopic operative removal of the foreign body which showed a 4 cm toothpick (Figure 2). Her postoperative course was uneventful and she recovered gradually. The patient was discharged home on postoperative Day 5 with Enoxaparin Subcutaneous 60 mg twice daily for 3 months.

### 2.6 Outcome and follow‐up

**FIGURE 3:**
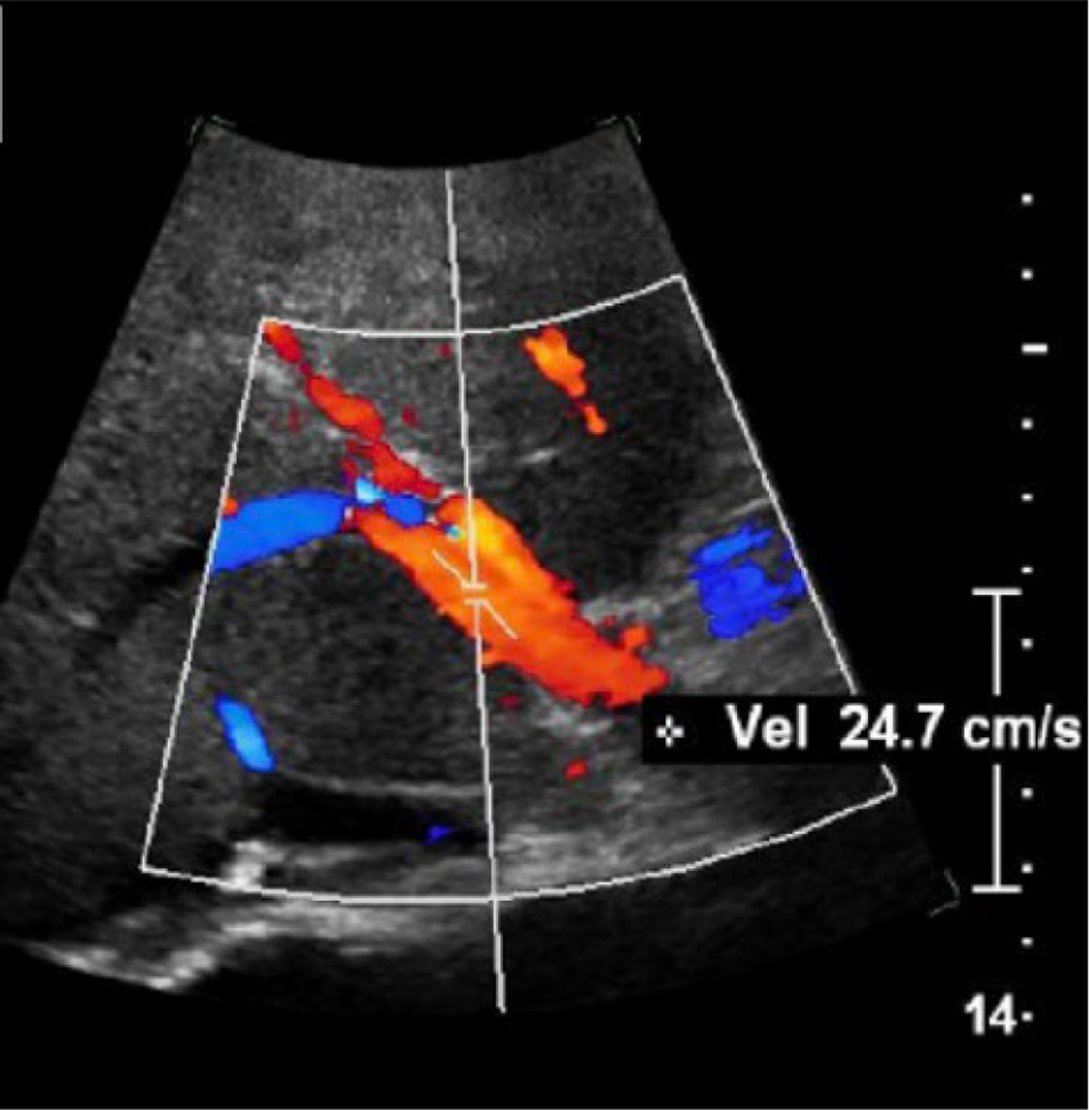
Doppler study of portal vein showed complete recanalization with normal blood flow.

After 2‐year follow‐up duration a liver doppler ultrasonography (US) was performed showing a patent portal vein with recanalization of the left branch without focal liver lesion (Figure 3).

## 3 DISCUSSION

Generally, hepatic abscess tends to form secondary to biliary tract diseases, infectious dissemination through the portal system, or hematological spread.^15^, ^16^, ^17^ Such an event the existence of a foreign body within the liver is rarely incidental in presentation. The majority of ingested objects pass through the alimentary canal uneventfully.^18^, ^19^ However, in a few cases, the ingested foreign bodies can penetrate the gastrointestinal tract and cause complications including gastrointestinal perforation or intra‐abdominal abscess.^18^, ^20^ In 1898, Lambert published the first case of hepatic abscess secondary to foreign body perforating the gastrointestinal tract.^13^ Since then, a migrated foreign body has been recognized as a cause of liver abscess treatment failure.^21^ Only 5%–8% of patients recall the ingestion of a foreign body in history.^20^ Analysis of the previous literature revealed some risk factors associated with toothpick ingestion: Male gender; habitual chewing of a toothpick; consuming food containing toothpicks.^22^ The mechanism by which the development of foreign body‐induced liver abscess is unclear.^23^ However, studies suggest the association of foreign object migration from the gut passing through the pylorus.^24^ Thus, the structural contraction of the pyloric sphincter can subsequently narrow the lumen, and given the close proximity of the anatomical location the penetration of the foreign body into the liver lobe can result in abscess formation.^24^ Liver abscess mainly occurs in the right lobe rather than the left lobe because of the path‐organism effect bringing the perfusion of hepatic portal flow.^24^ The clinical presentation of ingested foreign bodies is extremely heterogeneous and highly dependent on several factors including the time, the site, and the depth of the perforation, Furthermore, the symptoms range from mild abdominal pain to full‐blown septicemia.^18^, ^19^ The presentation is usually asymptomatic in most cases of foreign body ingestion and remains unnoticed. However, over time it develops with severely manifested signs of systemic infection, including abdominal pain, fever, nonresponsiveness to conservative management, intraabdominal abscess, and even shock if the foreign body perforates a major blood vessel.^19^, ^23^, ^25^ The mortality rate associated with toothpick ingestion is 18%.^26^ The diagnosis is challenging in such cases as the patient can present with vague symptoms and detecting the ingested foreign body on a plain radiography or computed tomography (CT) is difficult to appreciate due to their radiolucent nature which can contribute to delaying the diagnosis and treatment increasing the risk of mortality and morbidity.^18^, ^23^, ^27^ The diagnostic modality should be a high clinical index of suspicion, including a CT scan, US, upper GI endoscopy, and colonoscopy. A CT scan is made to confirm the diagnosis and plays a significant role in delineating the linear penetrated objects that are located within the liver. US can reveal the radio‐lucent foreign body^19^, ^24^ upper endoscopy is used in suspected penetration of the left lobe of the liver. A colonoscopy is used in suspected penetration to the right lobe of the liver. Al‐Khyatt et al.^28^ reported an incidental toothpick in the porta hepatic during a laparoscopic cholecystectomy.^28^ The management of foreign body‐induced liver abscess is by the removal of a foreign body by laparoscopic surgery, endoscopic procedure, or open surgery.^23^ The endoscopic procedure is used in cases when the foreign boy is located between the gastrointestinal tract and the liver.^23^ Surgical removal with adequate drainage is used when a foreign object penetrates the liver tissue.^23^ An open surgical approach is used in cases of deeply penetrated foreign objects in the liver parenchyma.^23^ Early removal of foreign objects and abscess drainage with adequate infection control are core‐stone management of migratory foreign body liver abscesses and necessitate a better fundamental treatment outcome.^24^

**FIGURE 4:**
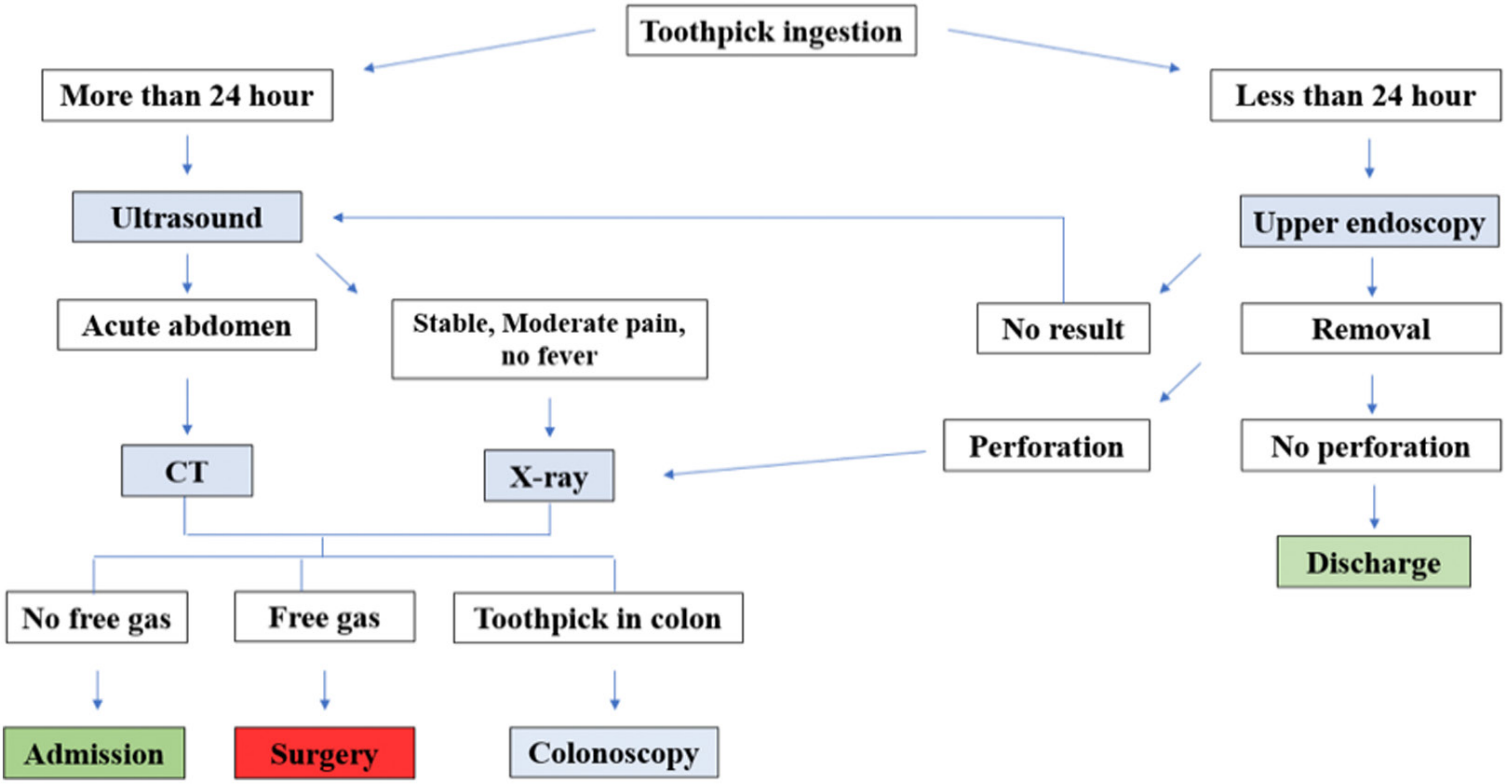
An algorithm summarizing the diagnostic and therapeutic guidelines used in cases of liver abscess complicated with portal vein thrombosis.

We report a case of a foreign body (toothpick) penetrating the gastric wall and migrating to the liver causing left hepatic abscess and portal vein thrombosis. To the best of our knowledge, there are no reported cases of portal vein thrombosis sequelae to foreign body migration causing hepatic abscess. The literature review was done in the PubMed database from 1950 to 2023 and only 28 cases of hepatic abscess secondary to toothpick were found, with the stomach as the most common site of penetration. Almost all cases were treated with antibiotics and removal of the foreign body either endoscopically or surgically with drainage of the abscess. Removal of toothpicks is generally done in all reviewed cases except for Chiang et al. where the patient was treated with IV antibiotics and showed significant improvement.^27^, ^29^, ^30^, ^31^, ^32^, ^33^, ^34^, ^35^, ^36^, ^37^, ^38^, ^39^, ^40^, ^41^, ^42^, ^43^, ^44^, ^45^, ^46^, ^47^ A discriptive algorithm summarizing the diagnostic and therapeutic guidelines (Figure 4).

## 4 CONCLUSION

The ingested foreign body can be a hidden cause of vague abdominal pain. The suspicion of an existing foreign body should be raised in awareness and a high index of suspicion for the possibility of an ingested foreign body should be implemented when a patient presents to the emergency department with unresolved abdominal pain.

## AUTHOR CONTRIBUTIONS

**Faisal Joueidi:** Data curation; writing – original draft; writing – review and editing. **Ali A. Alzahrani:** Data curation; supervision; writing – original draft; writing – review and editing. **Abdulaziz A. Altaweel:** Data curation; supervision; writing – original draft; writing – review and editing. **Omar Alwhaibi:** Data curation; writing – original draft; writing – review and editing. **Ahmed Elgohary:** Data curation; supervision; writing – review and editing. **Khalid O. Bin Saad:** Data curation; supervision; writing – review and editing.

## FUNDING INFORMATION

None.

## CONFLICT OF INTEREST STATEMENT

None to declare.

## ETHICS STATEMENT

The manuscript has been reviewed and approved by the IRB and Public Affairs Office.

## CONSENT

The authors have confirmed that patient consent has been signed and collected in accordance with the journal's patient consent policy.

## Data Availability

The corresponding author can provide the datasets analyzed in this study upon request. Additionally, the manuscript appropriately cites the resources used for the review and is readily available.
